# General Psychosomatic Medicine or the Loss of the Core of Being

**DOI:** 10.1192/j.eurpsy.2022.988

**Published:** 2022-09-01

**Authors:** S. Bast

**Affiliations:** German Academy for Psychoanalysis, Head Of Training Medical Doctors In Psychotherapy, Berlin, Germany

**Keywords:** Illness as “creative endeavour, fear equivalents, psychosomatic reaction

## Abstract

**Introduction:**

The authors presents an overview of the schools of learning in the area of modern psychosomatic medicine.

**Objectives:**

The author presents different variants for the concept of disease in psychosomatics.

**Methods:**

Groddeck was of the opinion that illness was a “creative endeavour”. Adler speaks of the will to be ill. Schulz Henke found that there are “gaps” where one would expect “normal life coping”. Heraclitus said the character of the human is his fate. In psychosomatic medicine, we must focus attention on the character failings. Viktor von Weizäcker spoke of the revolving door principle. Gebsattel concentrated on the inhibition in becoming. Arthur Jores described psychosomatic disorders as human illnesses. Humans become sick when they find themselves in a “dead-end-street of destiny”. They lose their core of being. Günther Ammon describes the psychosomatic reaction as the expression of a disturbed interaction process and advocates the psychoanalytical group therapy in the treatment of psychosomatic illnesses.

**Results:**

In psychosomatics one looks for a special personality type or for a special trigger situation. One asks about the childhood anamnesis and the biography, about the characteristic drives and the character problems for the respective illness.Those who have lost their core of being can regain it through self-education and self-reflection. However, a “core of being” must be present.

**Conclusions:**

Depending on the illness, character and social environment, it can happen that a patient “learns to express his wishes and fantasies, needs and sensitivities through his respective physical symptoms and complaints.

**Disclosure:**

No significant relationships.

